# Management, Surveillance Patterns, and Costs Associated With Low-Grade Papillary Stage Ta Non–Muscle-Invasive Bladder Cancer Among Older Adults, 2004-2013

**DOI:** 10.1001/jamanetworkopen.2022.3050

**Published:** 2022-03-18

**Authors:** Kelly K. Bree, Yong Shan, Patrick J. Hensley, Niyati Lobo, Chengrui Hu, Douglas S. Tyler, Karim Chamie, Ashish M. Kamat, Stephen B. Williams

**Affiliations:** 1 Department of Urology, University of Texas MD Anderson Cancer Center, Houston; 2 Division of Urology, Department of Surgery, University of Texas Medical Branch, Galveston; 3 Department of Surgery, University of Texas Medical Branch, Galveston; 4 Department of Urology, University of California, Los Angeles, Los Angeles

## Abstract

**Importance:**

Low-risk non–muscle-invasive bladder cancer (NMIBC) is associated with extremely low rates of progression and cancer-specific mortality; however, patients with low-risk NMIBC may often receive non–guideline-recommended and potentially costly surveillance testing and treatment.

**Objective:**

To describe current surveillance and treatment practices, cancer outcomes, and costs of care for low-grade papillary stage Ta (low-grade Ta) NMIBC and identify factors associated with increased cost of care.

**Design, Setting, and Participants:**

This population-based cohort study identified 13 054 older adults (aged 66-90 years) diagnosed with low-grade Ta tumors in the Surveillance, Epidemiology and End Results–linked Medicare database from January 1, 2004, through December 31, 2013. Medicare claims data through December 31, 2014, were also reviewed. Data were analyzed from April 1 to October 6, 2021.

**Exposures:**

Surveillance testing and treatment among patients with low-grade Ta NMIBC.

**Main Outcomes and Measures:**

The primary outcome was patterns in population-level surveillance and treatment practice over time among patients with low-grade Ta NMIBC. Secondary outcomes were recurrence (defined as receipt of subsequent transurethral resection of bladder tumor >3 months after index diagnosis of NMIBC and initial transurethral resection of bladder tumor), progression (defined as receipt of definitive treatment for bladder cancer), and costs of care.

**Results:**

Among 13 054 patients who met inclusion criteria, 9596 (73.5%) were male and 3458 (26.5%) were female, with a median age of 76 years (IQR, 71-81 years). A total of 403 patients (3.1%) were Black, 120 (0.9%) were Hispanic, 12 123 (92.9%) were White, and 408 (3.1%) were of other races and/or ethnicities. Rates of surveillance cystoscopy increased over the study period (from 79.3% in 2004 to 81.5% in 2013; *P* = .007), with patients receiving a median of 3.0 cystoscopies per year (IQR, 2.0-4.0 per year). Rates of upper tract imaging (particularly computed tomography or magnetic resonance imaging) also increased over the study period (from 30.4% in 2004 to 47.0% in 2013; *P* < .001), with most patients receiving a median of 2.0 imaging tests per year (IQR, 1.0-2.0 per year). The use of urine cytologic testing or other urine biomarker assessment also increased (from 44.8% in 2004 to 54.9% in 2013; *P* < .001). Rates of adherence to current guidelines were similar over time (eg, a median of 4398 patients [55.2%] received ≤2 cystoscopies per year in 2004-2008 vs a median of 2736 patients [53.8%] in 2009-2013; *P* = .11), suggesting overuse of all surveillance testing modalities. With regard to treatment, 2250 patients (17.2%) received intravesical bacillus Calmette-Guérin, and 792 patients (6.1%) received intravesical chemotherapy (excluding receipt of a single perioperative dose). Among all patients with low-grade Ta NMIBC, 217 (1.7%) experienced disease recurrence and 52 (0.4%) experienced disease progression. The total annual median costs of low-grade Ta surveillance testing and treatment increased by 60% (from $34 792 in 2004 to $53 986 in 2013), with higher 1-year median expenditures noted among those with disease recurrence ($76 669) vs no disease recurrence ($53 909) at the end of the study period.

**Conclusions and Relevance:**

In this cohort study, despite low rates of disease recurrence and progression, rates of surveillance testing increased during the study period. The annual cost of care also increased over time, particularly among patients with recurrent disease. Efforts to improve adherence to current practice guidelines, with the focus on limiting overuse of surveillance testing and treatment, may mitigate associated increasing costs of care.

## Key Points

**Question:**

What are the patterns in surveillance practices over time, oncologic outcomes, and costs associated with screening for low-grade papillary stage Ta non–muscle-invasive bladder cancer (NMIBC)?

**Findings:**

In this cohort study of 13 054 patients aged 66 to 90 years with a diagnosis of low-grade papillary Ta NMIBC in the Surveillance, Epidemiology, and End Results–linked Medicare database, significant increases in the use of cystoscopy, upper tract imaging, and urine cytologic testing occurred between 2004 and 2013. The annual median 1-year cost of care for patients with low-grade noninvasive disease increased by 60% over the study period (from $34 792 to $53 986) despite disease progression occurring in only 0.4% of patients.

**Meaning:**

These results suggest that, despite low rates of disease recurrence and progression in this patient population, efforts to limit overuse of surveillance testing and treatment are warranted to help mitigate increasing costs of care.

## Teaser

This cohort study uses data from the Surveillance, Epidemiology and End Results–linked Medicare database to describe current surveillance testing and treatment practices, cancer outcomes, and costs of care among older adults with low-grade papillary stage Ta non–muscle-invasive bladder cancer.

## Introduction

In 2021, it was estimated that approximately 63 000 new cases of non–muscle-invasive bladder cancer (NMIBC) would be diagnosed, and approximately 50% of those cases would involve low-grade papillary Ta (low-grade Ta) disease.^1,2^ Although NMIBC recurrence rates can be as high as 50% to 70% and progression rates as high as 10% to 30%,^2^ rates of progression are low among patients with low-grade Ta NMIBC. Among patients with low-grade Ta disease, the 15-year progression-free survival rate is 95%, with cancer-specific mortality rates less than 1%.^3,4^ Efforts have been made worldwide by the American Urological Association (AUA)/Society of Urologic Oncologists (SUO),^5^ the European Association of Urology,^6^ and the International Bladder Cancer Group^4^ to deescalate surveillance and treatment for those with low-grade Ta disease while maintaining appropriately frequent surveillance for those with high-grade aggressive disease.

Bladder cancer has the highest lifetime treatment cost of all cancers, with substantial economic burden throughout the entire disease course.^7^ Despite the high financial burden associated with bladder cancer and the low risk of progression and mortality associated with low-grade Ta disease (which comprises approximately 30 000 new cases in the US annually), a recent study of SUO members by Matulay et al^8^ reported overuse of upper tract imaging, cystoscopy, and urinary cytologic testing among low-risk patients. These diagnostic tests have been associated with patient morbidity and substantial financial toxic effects for the patient and the health care system.^9^ Thus, the aim of this cohort study was to describe population-based surveillance testing, treatment patterns, and costs of care for low-grade Ta NMIBC and identify factors associated with increased cost of care.

## Methods

### Data Source

This cohort study used data from the Surveillance, Epidemiology and End Results (SEER)–linked Medicare (SEER-Medicare) database. The SEER-Medicare database is sponsored by the National Cancer Institute and aggregates data from 18 defined geographic areas with 98% case ascertainment.^10^ The study was deemed exempt from review by the institutional review board of the University of Texas Medical Branch, and informed consent was waived because of the deidentified nature of the data. This study followed the Strengthening the Reporting of Observational Studies in Epidemiology (STROBE) reporting guideline for cohort studies.^11^

### Study Cohort

The study included older adults (aged 66-90 years) with a diagnosis of low-grade Ta urothelial bladder cancer between January 1, 2004, and December 31, 2013 (eFigure in the Supplement). Medicare claims data were reviewed through December 31, 2014. Data were analyzed from April 1 to October 6, 2021. We excluded patients older than 90 years, those with node-positive and/or metastatic disease, those with tumor stage of Tis/T1 or greater, those without continuous Medicare fee-for-service coverage, and those without available Medicare Part A and Part B claims data for 12 months before and after diagnosis.

### Identification of Surveillance Testing and Treatments

We identified patients who underwent cystoscopy, upper tract imaging, or urinary cytologic testing using *Current Procedural Terminology* codes (eTable 1 in the Supplement) from the Medicare claims data. Upper tract imaging modalities included intravenous pyelography, retrograde pyelography, kidney ultrasonography, abdominal and pelvic computed tomography (CT), and magnetic resonance imaging. Urine-based tests included urine cytologic testing or other urine biomarker assessment. We also assessed the treatments administered, including intravesical bacillus Calmette-Guérin (BCG), intravesical chemotherapy (defined as receipt of intravesical chemotherapy >30 days after receipt of transurethral resection of bladder tumor [TURBT] and excluding receipt of a single perioperative dose), radiotherapy, systemic chemotherapy, and radical cystectomy.

### Study Variables

From the SEER-Medicare database, we identified clinical and sociodemographic information, including patient age, sex, race and ethnicity (including Hispanic, non-Hispanic Black, non-Hispanic other race [which was not defined further], and non-Hispanic White), marital status, and geographic region of residence (based on US census regions). Educational level was defined according to the proportion of residents in the patient’s zip code who were older than 25 years and had received at least a high school diploma. Zip code–level median household income was used to define income quartiles. Both education and income information were obtained from the 2000 US census and the 2008 to 2012 American Community Survey.^12,13^ Comorbidities were identified through the Medicare claims data using the Klabunde modification of the Charlson Comorbidity Index during the year before cancer diagnosis.^14^

### Outcomes

The primary outcome of interest was patterns of surveillance testing, treatment, and cancer outcomes. Recurrence was defined as receipt of subsequent TURBT more than 3 months after index diagnosis of NMIBC (without progression) and initial transurethral resection of bladder tumor. Progression was defined as receipt of definitive treatment for bladder cancer (radical cystectomy, systemic chemotherapy, and/or radiotherapy). Adherence to current AUA/SUO clinical guidelines for low-risk NMIBC was also assessed.^5^ Patients were considered a priori to be adherent to guideline-recommended surveillance if they completed the following procedures within 1 year of diagnosis: (1) 2 or fewer cystoscopies, (2) 1 or no cytologic tests or urinary biomarker assessments, and (3) 1 or no upper tract imaging scans.

The secondary outcome was cost of low-grade Ta surveillance determined by Medicare claims data. Patients were followed up from the diagnosis date for 1 year or until death to assess the total costs of care. All Medicare expenditures (inpatient, outpatient, and physician services) within 1 year of diagnosis were summed to obtain total costs from the date of NMIBC diagnosis.^15^ Costs were stratified by disease recurrence status. All costs were inflated to 2020 US dollars using the Consumer Price Index for all urban consumers.^16^

### Statistical Analysis

Demographic and disease characteristics were summarized across all patients. Multivariable logistic regression models were used to examine variables associated with guideline-adherent surveillance. No variable selection was performed. Candidate covariates were determined a priori and included age at diagnosis (66-70 years, 71-75 years, 76-80 years, or 81-90 years), sex (male vs female), race and ethnicity (Black, Hispanic, White, or other race and/or ethnicity), geographic census region (Midwest, Northeast, South, or West), median household income (≤$42 992, $42 993-$56 188, $56 189-$73 827, or ≥$73 828), educational level (≤20.58%, 20.59%-27.36%, 27.37%-34.83%, or ≥34.84% of residents within the patient’s zip code with at least a high school diploma), comorbidities (Charlson Comorbidity Index score of 0, 1, 2, or ≥3), and year of diagnosis (2004-2008 vs 2009-2013). Exact values for covariates pertaining to fewer than 11 patients could not be reported in accordance with reporting guidelines from the SEER program. Because of the skewed nature of health care costs, medians and IQRs were used to describe 180-day and 365-day costs. Unadjusted median costs between patients with and without recurrence were compared using the Hodges-Lehmann estimator. All tests were 2-tailed and used a predetermined significance threshold of *P* < .05. Analyses were performed using SAS software, version 9.4 (SAS Institute Inc).

## Results

The study cohort consisted of 13 054 patients with low-grade Ta NMIBC (Table 1). A total of 9596 patients (73.5%) were male, and 3458 patients (26.5%) were female, with a median age of 76 years (IQR, 71-81 years). Overall, 403 patients (3.1%) were Black, 120 (0.9%) were Hispanic, 12 123 (92.9%) were White, and 408 (3.1%) were of other races and/or ethnicities. Most patients were married (7979 individuals [61.1%]) and had no or few comorbidities (9292 individuals [71.2%] with a Charlson comorbidity score of 0-1). The median follow-up duration was 84.8 months (IQR, 52.9-109.7 months).

**Table 1.** Baseline Characteristics of Patients With Low-Grade Stage Ta Non–Muscle-Invasive Bladder Cancer

Characteristic	Patients, No. (%)
Total patients, No.	13 054
Age at diagnosis, median (IQR), y	76 (71-81)
Sex	
Female	3458 (26.5)
Male	9596 (73.5)
Race and ethnicity	
Black	403 (3.1)
Hispanic	120 (0.9)
White	12 123 (92.9)
Other^a^	408 (3.1)
Marital status	
Single	1620 (12.4)
Married	7979 (61.1)
Unknown	3455 (26.5)
Census region	
West	4767 (36.5)
Midwest	3373 (25.8)
South	1464 (11.2)
Northeast	3450 (26.4)
Median household income, $	
≤42 992	3389 (26.0)
42 993-56 188	3182 (24.4)
56 189-73 827	3241 (24.8)
≥73 828	3242 (24.8)
High school educational level, %^b^	
≤20.58	3053 (23.4)
20.59-27.36	2995 (22.9)
27.37-34.83	3177 (24.3)
≥34.84	3829 (29.3)
Charlson Comorbidity Index score	
0	5814 (44.5)
1	3478 (26.6)
2	1750 (13.4)
≥3	2012 (15.4)
Year of diagnosis	
2004	1859 (14.2)
2005	1618 (12.4)
2006	1563 (12.0)
2007	1485 (11.4)
2008	1441 (11.0)
2009	1251 (9.6)
2010	925 (7.1)
2011	890 (6.8)
2012	981 (7.5)
2013	1041 (8.0)

^a^ Further details in this category were not available in the data source.

^b^ Educational level was based on the proportion of residents within a patient’s zip code who were older than 25 years with at least a high school diploma.

Most patients underwent cystoscopy, with rates increasing over time (79.3% of patients in 2004 to 81.5% of patients in 2013; *P* = .007). Patients underwent a median of 3.0 cystoscopies per year (IQR, 2.0-4.0 per year) after their diagnosis. Upper tract imaging was performed after diagnosis in most patients. The use of kidney ultrasonography (from 19.0% of patients in 2004 to 23.2% of patients in 2013) and retrograde pyelography (from 20.9% of patients in 2004 to 24.2% of patients in 2013) remained relatively stable over the study period. Use of intravenous pyelography decreased (from 14.5% of patients in 2004 to 1.7% of patients in 2012); however, there was a stepwise increase in the use of CT and magnetic resonance imaging in all years except 2010 (from 30.4% of patients in 2004 to 47.0% of patients in 2013; *P* < .001), with a median of 2.0 scans (IQR, 1.0-2.0 scans) performed per year. The rate of urine-based testing significantly increased during the study period (from 44.8% in 2004 to 54.9% in 2013; *P* < .001), with a median ranging from 2.0 to 3.0 tests per year (eg, 2.0 tests [IQR, 1.0-3.0] in 2004 to 3.0 tests [IQR, 2.0-6.0] in 2013). Additional patterns of surveillance testing among patients with low-grade Ta tumors are shown in Table 2.

**Table 2.** Surveillance Testing Among Patients With Low-Grade Stage Ta Non–Muscle-Invasive Bladder Cancer^a^

Year	Patients With NMIBC, No.	CT or MRI		Kidney ultrasonography		Retrograde pyelography		IV pyelography		Cystoscopy^b^		Urine cytologic or other urine biomarker test^c^	
		No. (%)	Median (IQR)	No. (%)	Median (IQR)	No. (%)	Median (IQR)	No. (%)	Median (IQR)	No. (%)	Median (IQR)	No. (%)	Median, IQR
2004	1859	566 (30.4)	2.0 (1.0-3.0)	353 (19.0)	1.0 (1.0-2.0)	389 (20.9)	2.0 (1.0-4.0)	270 (14.5)	2.0 (1.0-2.0)	1475 (79.3)	3.0 (2.0-4.0)	832 (44.8)	2.0 (1.0-3.0)
2005	1618	555 (34.3)	2.0 (1.0-3.0)	333 (20.6)	1.0 (1.0-2.0)	331 (20.5)	2.0 (1.0-3.0)	168 (10.4)	2.0 (1.0-2.0)	1273 (78.7)	3.0 (2.0-4.0)	835 (51.6)	2.0 (1.0-4.0)
2006	1563	588 (37.6)	2.0 (1.0-3.0)	298 (19.1)	1.0 (1.0-2.0)	311 (19.9)	2.0 (1.5-4.0)	124 (7.9)	2.0 (1.0-2.0)	1244 (79.6)	3.0 (2.0-4.0)	827 (52.9)	2.0 (1.0-4.0)
2007	1485	591 (39.8)	2.0 (2.0-4.0)	253 (17.0)	1.0 (1.0-2.0)	300 (20.2)	3.0 (1.5-4.0)	73 (4.9)	2.0 (1.0-2.0)	1148 (77.3)	3.0 (2.0-4.0)	769 (51.8)	2.0 (1.0-4.0)
2008	1441	594 (41.2)	2.0 (2.0-4.0)	290 (20.1)	1.0 (1.0-2.0)	300 (20.8)	2.0 (1.0-4.0)	80 (5.6)	2.0 (1.0-2.0)	1144 (79.4)	3.0 (2.0-3.0)	746 (51.8)	2.0 (1.0-4.0)
2009	1251	534 (42.7)	2.0 (2.0-4.0)	249 (19.9)	1.0 (1.0-2.0)	247 (19.7)	2.0 (1.0-3.0)	54 (4.3)	1.0 (1.0-2.0)	1010 (80.7)	3.0 (2.0-4.0)	675 (54.0)	3.0 (2.0-4.0)
2010	925	374 (40.4)	2.0 (2.0-4.0)	181 (19.6)	1.0 (1.0-2.0)	203 (21.9)	3.0 (2.0-4.0)	29 (3.1)	1.0 (1.0-2.0)	750 (81.1)	3.0 (2.0-3.0)	509 (55.0)	3.0 (2.0-4.0)
2011	890	382 (42.9)	2.0 (1.0-2.0)	173 (19.4)	1.0 (1.0-2.0)	198 (22.2)	2.0 (1.0-4.0)	24 (2.7)	2.0 (1.0-2.0)	727 (81.7)	3.0 (2.0-4.0)	515 (57.9)	3.0 (2.0-5.0)
2012	981	431 (43.9)	2.0 (1.0-2.0)	205 (20.9)	1.0 (1.0-2.0)	224 (22.8)	2.0 (2.0-4.0)	17 (1.7)	2.0 (1.0-2.0)	796 (81.1)	3.0 (2.0-4.0)	581 (59.2)	3.0 (2.0-6.0)
2013	1041	489 (47.0)	2.0 (1.0-2.0)	241 (23.2)	1.0 (1.0-2.0)	252 (24.2)	2.0 (2.0-4.0)	NR	2.0 (1.0-2.0)	848 (81.5)	3.0 (2.0-4.0)	571 (54.9)	3.0 (2.0-6.0)

Abbreviations: CT, computed tomography; IV, intravenous; MRI, magnetic resonance imaging; NR, not reported; NMIBC, non–muscle-invasive bladder cancer.

^a^ Cochran Armitage *P* < .001 for trend for all imaging modalities combined.

^b^ Cochran Armitage *P* = .007 for trend.

^c^ Cochran Armitage *P* < .001 for trend.

With regard to adherence to current guidelines for surveillance and treatment of low-risk NMIBC, a total of 4398 patients (55.2%) received 2 or fewer cystoscopies per year between 2004 and 2008 compared with a median of 2736 (53.8%) between 2009 and 2013 (*P* = .11). Rates of upper tract imaging increased over the study period (47.5% in 2004 to 49.2% in 2013; *P* = .06), and there was a significant increase in the number of patients who received more than 1 urine cytologic test per year (35.9% in 2004 to 46.0% in 2013; *P* < .001). Rates of induction and/or maintenance intravesical chemotherapy among patients with low-grade Ta disease also increased significantly over the study period (from 20.9% in 2004 to 23.1% in 2013; *P* = .003). However, the receipt of intravesical BCG decreased (18.5% in 2004 to 15.3% in 2013; *P* < .001). Additional rates of adherence to current low-risk NMIBC guidelines are available in eTable 2 in the Supplement.

Patient characteristics independently associated with more frequent cystoscopic evaluation (>2 cystoscopies per year) included female sex (odds ratio [OR], 0.70; 95% CI, 0.64-0.76), being married or having a partner (OR, 0.69; 95% CI, 0.61-0.77), and having a more recent NMIBC diagnosis (OR, 0.93; 95% CI, 0.86-1.00) (Table 3). Female sex (OR, 0.81; 95% CI, 0.74-0.88), being married or having a partner (OR, 0.83; 95% CI, 0.74-0.93), and having a more recent diagnosis (OR, 0.66; 95% CI, 0.61-0.71) were significantly associated with frequent cytologic testing (>1 test per year). Female sex (OR, 0.84; 95% CI, 0.77-0.91) was also independently associated with more frequent upper tract imaging (>1 test per year). Regional differences were also noted, with residence in the South and West being uniformly associated with more frequent surveillance testing (eg, receipt of ≤2 cystoscopies: OR, 0.70 [95% CI, 0.62-0.80] in the South and 0.80 [95% CI, 0.73-0.89] in the West). There were also patient-related factors associated with less frequent surveillance testing. Advanced age (81-90 years) was associated with less frequent cystoscopies (OR, 1.52; 95% CI, 1.36-1.69) and urine cytologic testing (OR, 1.14; 95% CI, 1.03-1.28), whereas Black race was associated with less frequent cystoscopies (OR, 1.41; 95% CI, 1.14-1.74) and upper tract imaging (OR, 1.36; 95% CI, 1.11-1.67). Patient characteristics associated with the receipt of intravesical BCG included Black race (OR, 0.75; 95% CI, 0.59-0.96) and being married or having a partner (OR, 0.82; 95% CI, 0.70-0.95). Black race (OR, 0.67; 95% CI, 0.54-0.84), residence in the South (OR, 0.81; 95% CI, 0.70-0.94), and more recent diagnosis (OR, 0.85; 95% CI, 0.78-0.93) were significantly associated with the receipt of intravesical induction and/or maintenance chemotherapy.

**Table 3.** Multivariable Analysis of Adherence to Current Screening and Treatment Guidelines

Variable	OR (95% CI)				
	≤2 Cystoscopies	≤1 Cytologic test	≤1 Upper tract imaging	Absence of BCG	Absence of chemotherapy^a^
Age range, y					
66-70	1 [Reference]	1 [Reference]	1 [Reference]	1 [Reference]	1 [Reference]
71-75	1.02 (0.92-1.14)	0.93 (0.83-1.03)	0.94 (0.85-1.05)	0.98 (0.86-1.13)	1.11 (0.98-1.27)
76-80	1.23 (1.10-1.37)	0.98 (0.87-1.09)	1.02 (0.91-1.14)	1.00 (0.87-1.16)	0.97 (0.86-1.11)
81-85	1.52 (1.36-1.69)	1.14 (1.03-1.28)	1.10 (0.99-1.23)	1.13 (0.98-1.30)	1.19 (1.05-1.35)
Sex					
Female	0.70 (0.64-0.76)	0.81 (0.74-0.88)	0.84 (0.77-0.91)	0.95 (0.85-1.06)	1.37 (1.23-1.51)
Male	1 [Reference]	1 [Reference]	1 [Reference]	1 [Reference]	1 [Reference]
Race and ethnicity					
Black	1.41 (1.14-1.74)	1.09 (0.88-1.35)	1.36 (1.11-1.67)	0.75 (0.59-0.96)	0.67 (0.54-0.84)
Hispanic	1.70 (1.14-2.53)	0.80 (0.55-1.17)	0.83 (0.58-1.19)	0.89 (0.56-1.41)	1.01 (0.65-1.56)
White	1 [Reference]	1 [Reference]	1 [Reference]	1 [Reference]	1 [Reference]
Other	1.13 (0.92-1.39)	0.88 (0.72-1.09)	0.85 (0.70-1.05)	0.80 (0.62-1.02)	0.86 (0.68-1.09)
Marital status					
Single	1 [Reference]	1 [Reference]	1 [Reference]	1 [Reference]	1 [Reference]
Married	0.69 (0.61-0.77)	0.83 (0.74-0.93)	0.91 (0.82-1.02)	0.82 (0.70-0.95)	1.03 (0.90-1.18)
Unknown	0.81 (0.72-0.92)	0.91 (0.81-1.04)	0.93 (0.82-1.05)	0.94 (0.79-1.11)	0.84 (0.73-0.98)
Census region					
Midwest	1 [Reference]	1 [Reference]	1 [Reference]	1 [Reference]	1 [Reference]
Northeast	1.40 (1.26-1.57)	0.54 (0.48-0.60)	0.84 (0.75-0.93)	1.11 (0.96-1.28)	1.17 (1.03-1.34)
South	0.70 (0.62-0.80)	0.53 (0.47-0.61)	0.63 (0.54-0.70)	1.65 (1.37-1.98)	0.81 (0.70-0.94)
West	0.80 (0.73-0.89)	0.80 (0.72-0.89)	0.56 (0.50-0.62)	1.02 (0.89-1.16)	1.33 (1.17-1.50)
Median household income, $					
≤42 992	1 [Reference]	1 [Reference]	1 [Reference]	1 [Reference]	1 [Reference]
42 993-56 188	0.92 (0.83-1.02)	0.89 (0.80-0.99)	0.94 (0.85-1.04)	0.95 (0.84-1.09)	1.02 (0.90-1.15)
56 189-73 827	0.89 (0.80-1.00)	0.80 (0.71-0.89)	0.96 (0.86-1.07)	1.01 (0.87-1.16)	1.13 (0.99-1.29)
≥73 828	0.86 (0.75-0.99)	0.77 (0.67-0.89)	1.01 (0.89-1.16)	1.01 (0.85-1.20)	1.05 (0.89-1.23)
High school educational level, %^b^					
≤20.58	1 [Reference]	1 [Reference]	1 [Reference]	1 [Reference]	1 [Reference]
20.59-27.36	0.99 (0.89-1.10)	1.09 (0.97-1.21)	1.13 (1.01-1.26)	0.87 (0.75-1.00)	0.90 (0.79-1.03)
27.37-34.83	1.04 (0.92-1.17)	1.20 (1.06-1.36)	1.03 (0.91-1.16)	0.83 (0.71-0.97)	0.91 (0.79-1.06)
≥34.84	1.01 (0.89-1.16)	1.25 (1.09-1.44)	0.97 (0.85-1.10)	0.87 (0.73-1.04)	0.88 (0.75-1.03)
Charlson Comorbidity Index score					
0	1 [Reference]	1 [Reference]	1 [Reference]	1 [Reference]	1 [Reference]
1	1.03 (0.95-1.12)	0.94 (0.86-1.03)	0.93 (0.85-1.01)	0.96 (0.86-1.07)	1.32 (1.19-1.46)
2	1.27 (1.14-1.42)	1.11 (0.99-1.24)	0.84 (0.75-0.94)	1.00 (0.87-1.16)	1.48 (1.29-1.70)
≥3	1.57 (1.41-1.75)	1.17 (1.05-1.30)	0.84 (0.76-0.94)	1.25 (1.08-1.44)	1.46 (1.28-1.66)
Year of diagnosis					
2004-2008	1 [Reference]	1 [Reference]	1 [Reference]	1 [Reference]	1 [Reference]
2009-2013	0.93 (0.86-1.00)	0.66 (0.61-0.71)	0.87 (0.91-1.04)	1.25 (1.13-1.38)	0.85 (0.78-0.93)

Abbreviations: BCG, bacillus Calmette-Guérin; OR, odds ratio.

^a^ Intravesical chemotherapy.

^b^ Educational level was based on the proportion of residents within a patient’s zip code who were older than 25 years with at least a high school diploma.

A total of 2250 patients (17.2%) received intravesical BCG, and 792 patients (6.1%) received chemotherapy (Table 4). Overall, 11 710 patients (89.7%) received at least 1 subsequent TURBT. A total of 217 patients (1.7%) experienced recurrence, and 52 patients (0.4%) experienced progression. Of those with disease progression, treatments included systemic chemotherapy (36 patients [0.3%]), radical cystectomy (<11 patients [0.1%]), and radiotherapy (<11 patients [0.1%]). There were 374 bladder cancer–specific deaths (2.9%) and 4392 deaths associated with other causes (33.6%).

**Table 4.** Treatments and Outcomes Among Patients With Low-Grade Stage Ta Non–Muscle-Invasive Bladder Cancer

Treatment or outcome	Patients, No. (%) (N = 13 054)
BCG	
No	10 804 (82.8)
Yes	2250 (17.2)
Intravesical chemotherapy^a^	
No	12 262 (93.9)
Yes	792 (6.1)
TURBT	
No	1344 (10.3)
Yes	11 710 (89.7)
Recurrence^b^	
No	12 837 (98.3)
Yes	217 (1.7)
Progression^c^	
No	13 002 (99.6)
Yes	52 (0.4)
Systemic chemotherapy	
No	13 018 (99.7)
Yes	36 (0.3)
Radical cystectomy	
No	>13 040 (99.9)
Yes	<11 (0.1)^d^
Radiotherapy	
No	>13 040 (99.9)
Yes	<11 (0.1)^d^
Death associated with bladder cancer	
No	12 680 (97.1)
Yes	374 (2.9)
Death associated with other causes	
No	8662 (66.4)
Yes	4392 (33.6)

Abbreviations: BCG, bacillus Calmette-Guérin; TURBT, transurethral resection of bladder tumor.

^a^ Beyond receipt of a single perioperative dose.

^b^ Includes any patient who received TURBT more than 3 months after index diagnosis without disease progression.

^c^ Defined as any patient who received definitive treatment for bladder cancer.

^d^ Exact values for variables pertaining to fewer than 11 patients could not be reported in accordance with reporting guidelines from the Surveillance, Epidemiology, and End Results program.

Total median costs at 1 year after diagnosis increased by 60% over the study period, from $34 792 in 2004 to $53 986 in 2013, with higher 1-year median expenditures noted among those with recurrence ($76 669) vs no recurrence ($53 909) at the end of the study period. Patients with disease recurrence had higher median 1-year costs at all time points (eg, median Hodges-Lehmann estimated difference in 2013: $24 313; 95% CI, −$2281 to $51 447), representing increases of up to 1.9-fold. Additional data on costs of care are shown in Table 5.

**Table 5.** Median 1-Year Medicare Costs After Diagnosis of Low-Grade Stage Ta Non–Muscle-Invasive Bladder Cancer

Year	Total median costs, $			Cost difference between recurrence vs no recurrence, Hodges-Lehmann estimate (95% CI), $
	All	Recurrence^a^	No recurrence	
180 Days after diagnosis				
2004	23 840	23 306	23 846	−1371 (−9754 to 7013)
2005	25 895	25 707	25 895	2784 (−5330 to 10 899)
2006	26 861	33 442	26 827	277 (−8440 to 8995)
2007	27 585	35 735	27 513	3301 (−6940 to 13 542)
2008	31 101	45 662	30 824	10 288 (−1047 to 21 622)
2009	30 475	30 611	30 301	−827 (−12 971 to 11 317)
2010	31 542	37 991	31 387	11 029 (−6842 to 28 901)
2011	34 249	36 939	34 249	2655 (−12 984 to 18 294)
2012	36 509	32 294	36 543	3206 (−12 796 to 19 208)
2013	38 499	48 151	38 463	2099 (−17 954 to 22 152)
365 Days after diagnosis				
2004	34 792	40 118	34 647	4820 (−8854 to 18 493)
2005	36 304	54 424	36 002	17 990 (4279 to 31 700)
2006	38 555	52 814	38 473	8780 (−4805 to 22 366)
2007	41 192	61 273	41 019	17 641 (2561 to 32 722)
2008	45 145	58 601	44 821	19 537 (7456 to 31 618)
2009	44 696	63 336	44 364	11 480 (−5815 to 28 776)
2010	46 198	98 029	46 032	43 608 (10 377 to 76 838)
2011	48 095	68 074	48 072	13 386 (−10 824 to 37 596)
2012	51 950	62 414	51 877	14 419 (−8828 to 37 665)
2013	53 986	76 669	53 909	24 313 (−2821 to 51 447)

^a^ Includes any patient who received transurethral resection of bladder tumor more than 3 months after index diagnosis without disease progression.

## Discussion

In this cohort study involving 13 054 patients with low-grade Ta NMIBC, frequent surveillance testing was common, and the intensity of surveillance increased over time. Moreover, treatments persisted, and cancer outcomes remained unchanged, with higher costs associated with management of recurrent disease. Among patients with low-grade Ta NMIBC, progression is rare, occurring in approximately 4% to 11% of patients.^3,17,18,19,20^ Notably, death rates are also negligible in this low-risk population.^3,17,19^ Given these favorable cancer outcomes, deescalation in surveillance among those with low-risk NMIBC compared with high-risk NMIBC is recommended by current international guidelines.^5,21^ However, consistent with the findings of the present study, practitioner-reported adherence to guidelines among low-risk patients remains low.^8^

Our study had several notable findings. First, to our knowledge, this study performed one of the largest population-based analyses of surveillance patterns among patients with low-grade Ta NMIBC. The data revealed that, despite the nonaggressive nature of low-risk low-grade Ta NMIBC, surveillance testing was performed frequently. We observed numerous patients receiving invasive procedures and urine-based tests, which are associated with morbidity and additional costs to the health care system, who experienced no change in cancer outcomes. Patients underwent a median of 3.0 cystoscopies per year after receiving their low-grade Ta diagnosis. Many patients also received a median of 2.0 CTs or magnetic resonance imaging scans and 2.0 to 3.0 urine-based tests, despite guidelines recommending against the use of these tests in the low-risk setting. Although these data predated current guidelines, risk stratification and early efforts to deescalate testing had been introduced during the study period; however, despite these changes, rates of testing increased over time. These findings are consistent with those of van Rhijn et al,^22^ which revealed low adherence to AUA/SUO treatment guidelines, particularly in North America, where adherence ranged from 0.5% to 29.0%. However, in contrast to other studies that primarily focused on the underuse of indicated treatments, such as intravesical BCG in the high-risk setting, the present study centered on overuse of testing in the low-risk patient population.

A previous study^23^ reported cancer outcomes and substantial costs among patients with high-risk NMIBC, and the present study added data that provide a more complete understanding of the entire spectrum of NMIBC, including observed surveillance patterns, overtreatment, and associated health care expenditures among patients with low-grade Ta disease. Our findings were consistent with those of Schroeck et al,^24^ who reported that frequent use of cystoscopy in the low-risk NMIBC setting was associated with a doubling in the number of TURBTs performed but was not associated with a decrease in the rates of progression or bladder cancer–specific mortality. Furthermore, Kukreja et al^25^ recently found that most patients who received surveillance cystoscopy reported moderate to severe discomfort and anxiety, highlighting the substantial patient morbidity associated with current screening practices.

Second, we found rates of surveillance testing significantly increased over the study period. Current AUA/SUO^5^ and International Bladder Cancer Group guidelines^4^ recommend 2 cystoscopies without upper tract imaging or urine cytologic testing during the first year after initial diagnosis of low-risk NMIBC. In the present study, use of all modalities of surveillance testing, including upper tract imaging and cytologic testing, increased over the study period. Since 2005, numerous international guidelines have advised deescalation in the use of surveillance cystoscopy, with no more than 3 cystoscopies recommended in the first 2 years after diagnosis; however, the findings of the present study suggest the use of cystoscopy has remained frequent and unchanged throughout the study period.^5,6,26^ These data also align with those from a Han et al^27^ study reporting overuse of cystoscopy in 75% of patients with low-risk NMIBC who were receiving care within the Department of Veterans Affairs. There is a paucity of data examining the use of upper tract imaging and urine-based testing in the low-risk NMIBC surveillance setting, but our results suggest that overuse of these surveillance tests may also be present.

Since 1999, clinical practice guidelines have been published and frequently updated to provide evidence-based guidance in the management of NMIBC.^5,6^ In 2007, risk stratification schemes were established^28^; however, it was not until 2016 that guidelines incorporated risk-stratified surveillance recommendations.^5^ The current study cohort reflected clinical practice patterns during the initiation of risk stratification but before formal guidance on the frequency and intensity of surveillance testing was available. It is plausible that rates of surveillance testing may have decreased after surveillance recommendations were published; however, we suspect that substantial changes in these rates were unlikely given the increase in testing despite risk stratification and historical data suggesting low adherence to guideline recommendations.^29,30^ It was also notable that among all of the surveillance tests performed, CT and magnetic resonance imaging had the most significant increase in use (1.6-fold) over the study period. In addition to the cost of these radiologic tests, CT urography, the most commonly performed CT imaging for upper tract surveillance, has been associated with at least a 1.5-fold increase in radiation dose compared with intravenous urography.^31^

Third, differences in surveillance and treatment patterns were also observed among certain patients with low-grade Ta NMIBC. Adults older than 81 years and Black individuals were more likely to experience deescalation in testing for their low-grade Ta tumors, whereas female patients were more likely to undergo testing. Differences in care delivery to adults with NMIBC who are older than 81 years have previously been described^32^; however, specific focus on surveillance practices among these older adults, especially given the increasing number of competing mortality risks with age, have not been investigated. Furthermore, although racial survivorship disparities among patients with NMIBC have been well established,^33^ most studies evaluating surveillance practices have had too few underrepresented minority participants to draw meaningful conclusions regarding racial and/or ethnic differences in surveillance practices. These data suggest that Black patients were more likely to receive deintensification of surveillance testing than their White counterparts, possibly owing to social factors associated with health in a setting with unequal access.^34^ The present study also found that female sex was associated with increased use of surveillance testing. Other research groups evaluating surveillance practices have not observed sex-specific differences; however, these studies were largely conducted within the Veterans Affairs system, in which there were relatively few female patients.^24,27^

Fourth, we described costs of care associated with low-grade Ta NMIBC. Health care expenditures within the US alone have continued to increase exponentially over the last several decades. It was estimated that $158 billion would be spent on direct medical costs in 2020, with more than 3% of all cancer-related costs being attributable to bladder cancer.^35^ In the present study, after accounting for inflation, the annual cost of low-grade Ta NMIBC increased by 60% over the study period, with increases of up to 1.9-fold among patients who experienced cancer recurrence. A previous group using SEER-Medicare data to evaluate the costs of early-stage bladder cancer reported that increased spending was largely associated with endoscopic surveillance.^36^ However, despite regional differences noted in bladder cancer spending and surveillance, no survival differences were noted.^36^ In the present cohort, despite the increasing prevalence of surveillance testing, rates of disease progression remained low at 0.4%, suggesting that rigorous surveillance testing was unlikely to provide substantial cancer benefit among patients with low-grade Ta disease. As we strive to improve bladder cancer care and health care spending, it will be important that clinicians be thoughtful about tests and procedures being performed. Goals might include delivery of risk-aligned surveillance that comprises more frequent surveillance of patients with high risk of disease progression and death as well as deescalation of surveillance among patients with low risk of worse cancer outcomes. The screening and treatment standards for other indolent urologic cancers, such as low-risk prostate cancer and small kidney tumors, have shifted substantially over the last decade. It is now time for patients with indolent low-grade Ta NMIBC to experience a similar change in disease management.

### Limitations

This study has several limitations. The findings should be interpreted within the context of the study design. First, patients included in this analysis were aged 66 to 90 years; thus, our findings may not be applicable to surveillance and treatment practices among younger patient populations. However, the mean age of NMIBC diagnosis is 73 years; thus, the present cohort is representative of a large proportion of patients with bladder cancer.^37^ Second, there are inherent limitations when using claims data, including potential inaccuracies in records and the inability to account for any treatments received outside of the Medicare system. Furthermore, the SEER-Medicare database lacks granular data on tumor staging and grading, which may impact the ability to accurately define patients with low-risk NMIBC using strict guideline criteria. Therefore, in accordance with previous studies,^38,39^ low-grade Ta NMIBC was used as a proxy to define low-risk NMIBC, recurrence, and progression.

Third, the study also had limitations in cancer end points. For example, it is possible that patients received TURBT for benign lesions, thereby producing overestimation of recurrence rates. It is also possible that patients experienced disease progression but did not receive definitive treatment, resulting in underestimation of rates of progression. Fourth, our analysis of guideline adherence retrospectively applies current guidelines to historical cohorts who received surveillance testing before formal guidelines were introduced, thereby preventing direct evaluation of adherence to guidelines. However, an overall pattern of increased surveillance testing was noted despite the implementation of risk stratification and efforts to deintensify surveillance during the study period.

Fifth, our analysis assumed that low-grade Ta tumors were small primary lesions and could therefore be categorized as low risk. However, multifocal recurrent low-grade Ta tumors are generally classified as intermediate risk and would thus be subject to more frequent surveillance testing than their low-risk counterparts; patients with these tumors would also be more likely to receive adjuvant therapies, resulting in overestimation of nonadherence to guidelines. Nevertheless, given the minimal risk of progression and disease-specific mortality, initial and recurrent low-grade Ta tumors can likely be managed safely through deescalated treatment and surveillance protocols similar to those proposed by the International Bladder Cancer Group.^4^

Sixth, inaccuracies in death classification may also have affected the unexpectedly high rate of bladder cancer–specific mortality in this cohort. Moghanaki et al^36^ previously found that the National Death Index provided accurate dates of death but frequently misclassified cause of death among patients with prostate cancer. It is plausible that similar misclassifications occurred among patients with bladder cancer. Nevertheless, it should be noted that rates of other causes of death remained more than 10 times higher than bladder cancer–specific deaths in this patient population, highlighting the relatively low mortality risk of bladder cancer compared with other diseases in this patient population. Seventh, our cost expenditures were based on previously accepted methods to capture total Medicare costs and may underestimate the costs of bladder cancer care in the non-Medicare population.^40,41^

## Conclusions

In this cohort study, patients with low-grade Ta NMIBC had low rates of recurrence, progression, and bladder cancer–specific death. Current clinical practice guidelines recommend deescalation in surveillance. The findings of the present study revealed that, despite guideline recommendations, frequent surveillance testing was common and was associated with increases in the annual cost of care over time. These data suggest a need for ongoing efforts to limit overuse of treatment and surveillance, which may in turn mitigate associated increases in the costs of care.

## Notes

Supplement. **eTable 1.** Drug and Procedure Codes **eTable 2.** Adherence to Current Low-Risk NMIBC Guidelines 1 Year After Diagnosis **eFigure.** Study Flow Diagram Illustrating Cohort Selection
