# Supplementary material for: Management, Surveillance Patterns, and Costs Associated With Low-Grade Papillary Stage Ta Non–Muscle-Invasive Bladder Cancer Among Older Adults, 2004-2013
Source: JAMA Netw Open. 2022 Mar 18;5(3):e223050. doi: 10.1001/jamanetworkopen.2022.3050 (PMC8933744; doi:10.1001/jamanetworkopen.2022.3050)
Supplement: Supplement. — eTable 1. Drug and Procedure Codes eTable 2. Adherence to Current Low-Risk NMIBC Guidelines 1 Year After Diagnosis eFigure. Study Flow Diagram Illustrating Cohort Selection [file jamanetwopen-e223050-s001.pdf]

Supplementary Online Content

Bree KK, Shan Y, Hensley PJ, et al. Management, surveillance patterns, and costs associated with low-grade papillary stage Ta non–muscle-invasive bladder cancer among older adults, 2004-2013. *JAMA Netw Open*. 2022;5(3):e223050. doi:10.1001/jamanetworkopen.2022.3050

eTable 1. Drug and Procedure Codes

eTable 2. Adherence to Current Low-Risk NMIBC Guidelines 1 Year After Diagnosis

eFigure. Study Flow Diagram Illustrating Cohort Selection

This supplementary material has been provided by the authors to give readers additional information about their work.

© 2022 Bree KK et al. *JAMA Network Open*.

eTable 1. Drug and Procedure Codes

	**CPT/HCPCS**	**ICD-9-PCS**
**Screening**		
**Cystoscopy**	52000	
**CT/MRI**	74176, 74177, 74178, 74181, 74182, 74183, 74150, 74160, 74170, 72194, 72197	
**Renal Ultrasound**	76770, 76775	
**IV Pyelography**	74400-74415	
**Retrograde pyelogram**	74420, 52005	
**Urine cytology or other urine biomarker**	88112, 88120, 88121, 86316, 86294, 86386, 81479, 88106, 88108	
**Treatment**		
**TURBT**	52234, 52235, 52240	
**BCG therapy**	J9031, 90586	
**Intravesical chemotherapy**	51720	
**Radical cystectomy**	51570, 51575, 51580, 51585, 51590, 51595, 51596, 51597	57.71, 57.79, 68.8
**Radiation**	77261, 77385, 77386, 77399, 77401-77499, 77371-77373, G0173, G0174, G0251, G0339, G0340, G6001-G6017, 77520-77525	92.21–92.29

Abbreviations: BCG, bacillus Calmette-Guérin; CPT, Current Procedural Terminology; CT, computed tomography; HCPCS, Healthcare Common Procedure Coding System; ICD-9(10)-PCS, International Classification of Diseases, Ninth (Tenth) Revision, Procedure Coding System; MRI, magnetic resonance imaging; TURBT, transurethral resection of bladder tumor.

© 2022 Bree KK et al. *JAMA Network Open*.

eTable 2. Adherence to Current Low-Risk NMIBC Guidelines 1 Year After Diagnosis[a]

Quality of care measure	2004-2008	2009-2013	
	N (%)	N (%)	P-value
**≤2 Cystoscopies**	4,398 (55.2)	2,736 (53.8)	0.108
**≤1 Cytology**	5,103 (64.1)	2,748 (54.0)	<.0001
**≤1 Upper tract imaging**	4,181 (52.5)	2,585 (50.8)	0.061
**Absence of any BCG instillation**	6,492 (81.5)	4,312 (84.7)	<.0001
**Absence of intravesical chemotherapy[b]**	6,302 (79.1)	3,912 (76.9)	0.0027

Abbreviations: BCG, bacillus Calmette-Guérin; NMIBC, non-muscle invasive bladder cancer.

[a]The AUA/SUO guidelines recommend the following surveillance within 1 year of diagnosis[5]:
- Cystoscopies: two (first-look cystoscopy at 3 months, followed by repeat cystoscopy at 6-9 months)
- Upper tract imaging: none
- Urine-based tests: none

[b]Intravesical chemotherapy: receipt of intravesical treatment beyond single perioperative dose

© 2022 Bree KK et al. *JAMA Network Open*.

eFigure. Study Flow Diagram Illustrating Cohort Selection

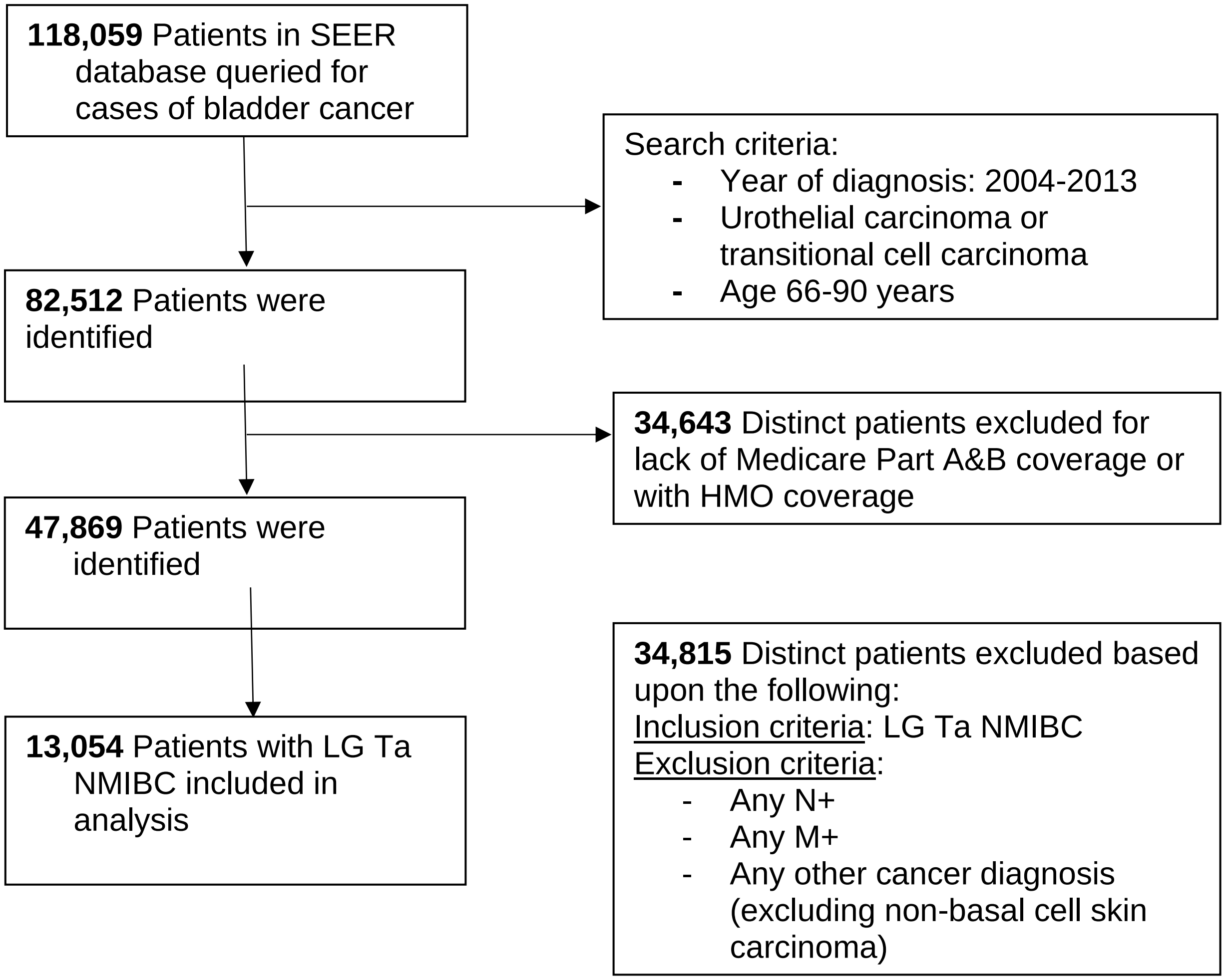

Abbreviations: HMO, health maintenance organization; NMIBC, non-muscle invasive bladder cancer; SEER, surveillance, epidemiology, and end results.

© 2022 Bree KK et al. *JAMA Network Open*.
